# Supplementary material for: Reaction Networks as Systems for Resource Allocation: A Variational Principle for Their Non-Equilibrium Steady States
Source: PLoS One. 2012 Jul 16;7(7):e39849. doi: 10.1371/journal.pone.0039849 (PMC3397975; doi:10.1371/journal.pone.0039849)
Supplement: Table S1 — Metabolites (abbreviation, full names, estimated concentrations) appearing in the reduced model of hRBC metabolism. (PDF) [file pone.0039849.s001.pdf]

Supporting Information for

Reaction networks as systems for resource allocation: a variational principle for their non-equilibrium steady states

Andrea De Martino, Daniele De Martino, Roberto Mulet, Guido Uguzzoni

Supporting Table S1

Abbreviation	Compound name	c [M]
GLC*	Glucose	$5 \pm 1 \cdot 10^{-3}$
G6P	Glucose-6-phosphate	$4 \pm 1 \cdot 10^{-5}$
F6P	Fructose-6-phosphate	$1.3 \pm 0.5 \cdot 10^{-5}$
FDP	Fructose-1,6-diphosphate	$2.7 \pm 1 \cdot 10^{-6}$
DHAP	Dihydorxyacetone phosphate	$1.7 \pm 0.1 \cdot 10^{-5}$
GA3P	Glyceraldehyde-3-phosphate	$5.7 \pm 1 \cdot 10^{-6}$
13DPG	1,3-Diphosphoglycerate	$1 \pm 0.5 \cdot 10^{-6}$
23DPG	2,3-Diphosphoglycerate	$4 \pm 3 \cdot 10^{-3}$
3PG	3-Phosphoglycerate	$4 \pm 2 \cdot 10^{-5}$
2PG	2-Phosphoglycerate	$1.4 \pm 0.5 \cdot 10^{-5}$
PEP	Phosphoenolpyruvate	$1.7 \pm 0.2 \cdot 10^{-5}$
PYR	Pyruvate	$8 \pm 6 \cdot 10^{-5}$
LAC*	Lactate	$1.4 \pm 0.5 \cdot 10^{-3}$
6PGL	6-Phosphogluco-lactone	
6PGC	6-Phosphogluconate	$5 \pm 2 \cdot 10^{-6}$
RL5P	Ribulose-5-phosphate	
X5P	Xylusose-5-phosphate	
R5P	Ribose-5-phosphate	
S7P	Sedoheptulose-7-phosphate	
E4P	Erythrose-4-phosphate	$5 \pm 2 \cdot 10^{-5}$
ADP	Adenosine diphosphate	$3 \pm 0.1 \cdot 10^{-4}$
ATP	Adenosine triphosphate	$3 \pm 0.1 \cdot 10^{-3}$
NAD	Nicotinamide adenine dinucleotide	$7 \pm 2 \cdot 10^{-5}$
NADH	Nicotinamide adenine dinucleotide(R)	around 10^{-7} (ext)
NADP	Nicotinamide adenine dinucleotide phosphate	$3 \pm 0.5 \cdot 10^{-5}$
NADPH	Nicotinamide adenine dinucleotide phosphate (R)	$6 \pm 2 \cdot 10^{-5}$
H*	Hydrogen ion	$10^{-7.2}$
Pi*	Inorganic phosphate	$1.0 \pm 0.5 \cdot 10^{-3}$
CO_2^*	Carbon dioxide	$1.2 \pm 2 \cdot 10^{-2}$
H_2O^*	Water	solvent

Metabolites appearing in the reduced model of hRBC metabolism. For reference, we include their estimated intracellular concentrations (when available; data were extracted from the BioNumbers database, see Milo R, Jorgensen P, Moran U, Weber G, Springer M (2010) BioNumbers–the database of key numbers in molecular and cell biology. Nucl. Acids Res. **38** (suppl. 1): D750–D753). The compounds marked with an asterisk can be subject to uptakes.
