# Supplementary material for: Reaction Networks as Systems for Resource Allocation: A Variational Principle for Their Non-Equilibrium Steady States
Source: PLoS One. 2012 Jul 16;7(7):e39849. doi: 10.1371/journal.pone.0039849 (PMC3397975; doi:10.1371/journal.pone.0039849)
Supplement: Table S2 — Reactions (abbreviation, enzyme name, formula) appearing in the reduced model of hRBC metabolism. (PDF) [file pone.0039849.s002.pdf]

Supporting Information for

Reaction networks as systems for resource allocation: a variational principle for their non-equilibrium steady states

Andrea De Martino, Daniele De Martino, Roberto Mulet, Guido Uguzzoni

Supporting Table S2

Nr	Abbr	Enzyme	reaction
1	HK	Hexokinase	$GLC + ATP \rightarrow G6P + ADP + H$
2	PGI	Phosphoglucoisomerase	$G6P \leftrightarrow F6P$
3	PFK	Phosphofructokinase	$F6P + ATP \rightarrow FDP + ADP + H$
4	ALD	Aldolase	$FDP \leftrightarrow GA3P + DHAP$
5	TPI	Triose phosphate isomerase	$DHAP \leftrightarrow GA3P$
6	GAPDH	GLyceraldehyde phosphate dhydrogenase	$GA3P + NAD + Pi \leftrightarrow 13DPG + NADH + H$
7	PGK	Phosphoglycerate kinase	$13DPG + ADP \leftrightarrow 3PG + ATP$
8	DPGM	Diphosphoglyceromutase	$13DPG \rightarrow 23DPG + H$
9	DPGase	Diphosphoglycerate phosphatase	$23DPG + H_2O \rightarrow 3PG + Pi$
10	PGM	Phosphoglyceromutase	$3PG \leftrightarrow 2PG$
11	EN	Enolase	$2PG \leftrightarrow PEP + H_2O$
12	PK	Pyruvate kinase	$PEP + ADP + H \rightarrow PYR + ATP$
13	LDH	Lactate dehydrogenase	$PYR + NADH + H \leftrightarrow LAC + NAD$
14	G6PDH	Glucose-6-phosphate dehydrogenase	$G6P + NADP \rightarrow 6PGL + NADPH + H$
15	PGL	6-phosphoglyconolactonase	$6PGL + H_2O \leftrightarrow 6PGC + H$
16	PDGH	6-phosphoglycoconate dehydrogenase	$6PGC + NADP \rightarrow RL5P + NADPH + CO_2$
17	R5PI	Ribose-5-phosphate isomerase	$RL5P \leftrightarrow R5P$
18	X5P	Xylulose-5-phosphate epimerase	$RL5P \leftrightarrow X5P$
19	TKI	Transketolase I	$X5P + R5P \leftrightarrow S7P + GA3P$
20	TA	Transaldolase	$GA3P + S7P \leftrightarrow E4P + F6P$
21	TKII	Transketolase	$X5P + E4P \leftrightarrow F6P + GA3P$
22	ATPase	Na-K pump	$ATP + H_2O \rightarrow ADP + Pi$
23	NADPHase	Glutathione reductase	$NADPH \rightarrow NADP + H$

Reactions appearing in the reduced model of hRBC metabolism. Processes 1–13 belong to glycolysis, 14–21 to the pentose-phosphate pathway; 22 and 23 are instead the pumps. Standard reversibility assignments (based on thermodynamic information) are represented by the arrows.
